# Influence of cirrhosis on long-term prognosis after surgery in patients with combined hepatocellular-cholangiocarcinoma

**DOI:** 10.1186/s12876-017-0584-y

**Published:** 2017-02-10

**Authors:** Yan-Ming Zhou, Cheng-Jun Sui, Xiao-Feng Zhang, Bin Li, Jia-Mei Yang

**Affiliations:** 1 Department of Hepatobiliary & Pancreatovascular Surgery, First affiliated Hospital of Xiamen University, Xiamen, China; 2 Department of Special Treatment, Eastern Hepatobiliary Surgery Hospital, Second Military Medical University, Shanghai, China

**Keywords:** Combined hepatocellular-cholangiocarcinoma, Long-term survival, Cirrhosis, Surgical resection

## Abstract

**Background:**

Little is known about the prognostic impact of cirrhosis on long-term survival of patients with combined hepatocellular-cholangiocarcinoma (cHCC-CC) after hepatic resection. The aim of this study was to elucidate the long-term outcome of hepatectomy in cHCC-CC patients with cirrhosis.

**Methods:**

A total of 144 patients who underwent curative hepatectomy for cHCC-CC were divided into two groups: cirrhotic group (*n* = 91) and noncirrhotic group (*n* = 53). Long-term postoperative outcomes were compared between the two groups.

**Results:**

Patients with cirrhosis had worse preoperative liver function, higher frequency of HBV infection, and smaller tumor size in comparison to those without cirrhosis. The 5-year overall survival rate in cirrhotic group was significantly lower than that in non-cirrhotic group (34.5% versus 54.1%, *P* = 0.032). The cancer recurrence-related death rate was similar between the two groups (46.2% versus 39.6%, *P* = 0.446), while the hepatic insufficiency-related death rate was higher in cirrhotic group (12.1% versus 1.9%, *P* = 0.033). Multivariate analysis indicated that cirrhosis was an independent prognostic factor of poor overall survival (hazard ratio 2.072, 95% confidence interval 1.041–4.123; *P* = 0.038).

**Conclusions:**

The presence of cirrhosis is significantly associated with poor prognosis in cHCC-CC patietns after surgical resection, possibly due to decreased liver function.

## Background

Combined hepatocellular-cholangiocarcinoma (cHCC-CC) is a very rare entity that includes elements of both hepatocellular carcinoma (HCC) and cholangiocarcinoma (CC) and represents 0.4–14.2% of primary liver malignancies [1]. Hepatic resection affords the best chance of long-term survival with a reported 5-year overall survival (OS) rate of 23.1–54.1%. Vascular invasion, lymph node metastasis, satellite nodules, and tumor size were reported as prognostic factors [2–5].

Patients with cHCC-CC, especially in Asian countries, are frequently accompanied by liver cirrhosis, with a prevalence of 27.7–84.6% [6]. However, little is known about the prognostic significance of cirrhosis in cHCC-CC patients after surgery. In this study, we compared the long-term outcomes of hepatic resection in cHCC-CC patients with and without cirrhosis.

## Methods

### Patients

From February 2000 to December 2011, 151 patients with cHCC-CC who underwent curative resection at our institutes. Curative resection was defined as complete excision of the tumor with clear microscopic margin conformed by histopathological examination. Allen and Lisa [7] categorized cHCC-CC into three types; type A: HCC and CC exist separately (double cancer); type B: HCC and CC exist contiguously but independentlyonly; and type C: HCC and CC components show contiguity with intermingling. Histologically, only type C tumors that displayed the characteristics of a genuine mixture of both HCC and CC elements were regarded as true combined tumors [5]. Seven patients with Allen type A and B tumors were therefore excluded from the study. Finally, 144 patients were subjected to this study. Of them, 91 (63.2%) patients had cirrhosis as confirmed by histology and the remaining 53 (36.8%) patients did not have cirrhosis. Patient demographics, operative data, tumor characteristics, and follow-up findings were reviewed retrospectively. Postoperative morbidity and mortality were analyzed 90 days after operation. Liver dysfunction was defined as total bilirubin level >10 mg/dL unrelated to biliary obstruction or leak and/or the international normalized ratio >2 for more than 2 days after resection and/or clinically significant ascites/hepatic encephalopathy [8].

All patients were followed postoperatively by serum tumor marker (alpha-fetoprotein [AFP] and carbohydrate antigen 19–9 [CA 19–9]) analysis and ultrasound or computed tomography at least every 3 months in the first year after hepatectomy, and then at gradually increasing intervals. Intrahepatic recurrence was identified by new lesions on imaging with typical appearances of cHCC-CC with or without a rising serum AFP or CA 19–9 level. Determination of treatment strategy for recurrent tumors depended on the number and site of the tumors, any concurrent extrahepatic recurrence, liver function, and the general status of the patient. Re-hepatectomy and percutaneous radiofrequency ablation (PRFA) were considered as first-choice treatments. Re-hepatectomy was performed for Child A patients with solitary or multiple tumors limited in the semi-liver with sufficient liver remnant volume. PRFA was given to Child A and selected Child B patients with solitary tumor ≦3 cm located deeply in the liver parenchyma or multiple tumors (up to 3 lesions all ≦ 3 cm) in different lobes without vascular invasion or gross ascites. Transarterial chemoembolization (TACE) was considered when the above two treatments were not possible, as in patients with advanced multinodular recurrent tumors, poor liver function, and insufficient liver remnant volume. Systemic chemotherapy or conservative treatment was considered for patients with extensive systemic recurrence and/or very poor liver function or general condition.

### Statistical analysis

Categorical and continuous data were compared by the *χ* ^2^ test and the Student *t* test, respectively. Patient OS and disease-free survival (DFS) rates were estimated using the Kaplan-Meier method, and differences between groups were compared by log-rank test. Multivariate analysis was performed by the Cox proportional hazard regression model. All statistical analyses were performed using SPSS for Windows (version 11.0; SPSS Institute, Chicago, IL, USA). *P* < 0.05 was considered statistically significant.

## Results

### Patient characteristics and outcomes

The clinicopathologic data of noncirrhotic and cirrhotic patients are summarized in Table 1. Cirrhotic patients had higher prevalence of men, alcohol abuse, and positive hepatitis B surface antigen (HBsAg), higher serum alanine aminotransferase (ALT) and aspartate aminotransferase (AST) levels, higher prevalence of abnormal serum AFP level, and smaller tumors than non-cirrhotic patients.

**Table 1:** Comparison of clinicopathologic features

Variables	Cirrhosis *n* = 91	Non- cirrhosis *n* = 53	*P*-value
Sex (male/female), *n*	89/2	46/7	0.274
Age (years; mean ± SD)	53.2 ± 9.2	52.1 ± 8.1	0.463
Overweight (BMI 25.0-29.99 kg/m^2^), *n* (%)	15 (16.5)	10 (18.9)	0.716
Obesity (BMI ≥ 30 kg/m2), *n* (%)	3 (3.3)	2 (3.8)	0.880
Hypertension, *n* (%)	12 (13.2)	6 (11.3)	0.744
Diabetes mellitus, *n* (%)	10 (11.0)	4 (7.5)	0.501
Hepatitis B surface antigen, *n* (%)	79 (63.8)	22 (37.2)	<0.001
Alcohol use, *n* (%)	24 (26.4)	5 (9.4)	0.015
Total bilirubin (μmol/L; mean ± SD)	17.8 ± 8.8	15.4 ± 4.1	0.090
Albumin (g/L; mean ± SD)	40.5 ± 5.3	41.2 ± 4.6	0.417
Aspartate aminotransferase (IU/L; mean ± SD)	51.2 ± 35.3	39.6 ± 22.3	0.021
Alanine aminotransferase (IU/L; mean ± SD)	54.5 ± 50.6	41.8 ± 36.7	0.043
Child-Pugh (A/B), *n*	85/6	53/0	0.056
Tumor diameter (cm; mean ± SD)	4.9 ± 2.5	6.7 ± 2.8	<0.001
Tumor number (St/Mt), *n*	82/9	46/7	0.541
Encapsulation, *n* (%)	34 (37.4)	15 (28.3)	0.268
Vascular invasion, *n* (%)	41 (45.1)	27 (50.9)	0.495
Bile duct invasion, *n* (%)	17 (18.7)	11(20.8)	0.762
Lymph node involvement, *n* (%)	12 (13.2)	8 (15.1)	0.750
Alpha-fetoprotein ≥ 20 ng/mL, *n* (%)	56 (61.5)	22 (41.5)	0.020
Carbohydrate antigen 19–9 ≥ 37 U/mL, *n* (%)	31 (34.1)	21 (39.6)	0.503
Carcinoembryogenic antigen ≥ 5 ng/mL, *n* (%)	7 (7.7)	3 (5.7)	0.644

*BMI* body mass index; *St* single tumor; *Mt* multiple tumors

Regarding operative procedures and preoperative outcomes, less major resection (≥3 segments) was applied in cirrhotic patients. Postoperative morbidity was similar in the two groups except for the higher incidence of liver dysfunction in cirrhotic group. One patient in cirrhotic group died of hepatorenal failure resulting in a mortality rate of 1.1%, showing no statistically significant difference with 0% in non-cirrhotic group (Table 2).

**Table 2:** Comparison of operative procedures and preoperative outcomes

Variables	Cirrhosis *n* = 91 (%)	Non- cirrhosis *n* = 53 (%)	*P*-value
Extent of resection			0.006
Major resection	21 (23.1)	24 (45.3)	
Minor resection	70 (76.9)	29 (54.7)	
Liver disfuction	31 (34.1)	7 (13.2)	0.006
Complications other than liver disfuction	34 (37.4)	18 (34.0)	0.682
Mortality	1 (1.1)	0 (0)	0.444

**Fig. 1:**
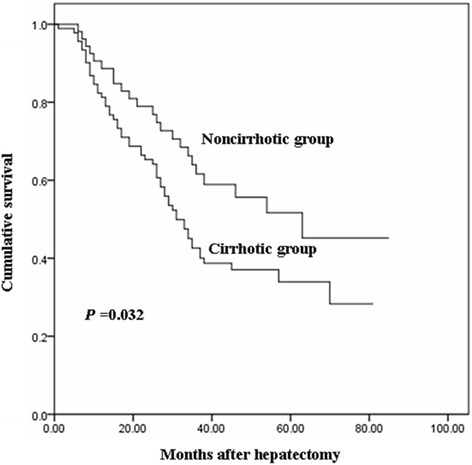
Comparison of patient overall survival rates between the cirrhotic and non-cirrhotic groups

The median postoperative follow-up period was 35 (range 3–127) months. The 5-year DFS rate was similar between cirrhotic and non-cirrhotic patients (29.6% *versus* 38.7%, *P* = 0.079). However, the 5-year OS rate and the median OS time in cirrhotic group was significantly lower than that in non-cirrhotic group, with values of 34.5% and 31 months, *versus* 54.1% and 63 months, respectively (*P* = 0.032) (Fig. 1).

By the time of analysis, recurrences developed in 68 cirrhotic and 35 non-cirrhotic patients with a similar frequency (75.5% *versus* 66.1%, *P* = 0.567). Also, there was no difference in the median time to recurrence and the pattern of recurrence between the two groups. Regarding the initial treatment for recurrences, aggressive approaches including re-hepatectomy and local ablation were applied less frequently in cirrhotic patients as compared with non-cirrhotic patients (36.8% *versus* 60.0%, *P* = 0.025) (Table 3).

**Table 3:** Tumor recurrence data

Variables	Cirrhosis *n* = 68	Non- cirrhosis *n* = 35	*P*-value
Median time to recurrence, months	13	14	0.693
Recurrence type, *n* (%)			
Intrahepatic recurrence	42 (61.8)	24 (68.6)	0.495
Extrahepatic recurrence	19 (27.9)	8 (22.8)	0.578
Both	7 (10.3)	3 (8.6)	0.780
Treatment of recurrence, *n* (%)			
Aggressive approach	25 (36.8)	21 (60.0)	0.025
Rehepatectomy	3 (4.3)	5 (14.3)	0.076
Local ablation	22 (32.4)	16 (45.7)	0.183
Transarterial chemoembolization	26 (38.2)	9 (25.7)	0.204
Systemic chemotherapy	5 (7.4)	2 (5.7)	0.754
Conservative treatment	12 (17.6)	3 (8.6)	0.216

Investigation on the cause of death showed that 56 cirrhotic patients and 23 non-cirrhotic patients died during the follow-up period in this study (*P* = 0.029). Cancer recurrence-related death was similar between cirrhotic and non-cirrhotic group (46.2% *versus* 39.6%, *P* = 0.446), while hepatic insufficiency-related death was more frequently observed in cirrhotic group (12.1% *versus* 1.9%, *P* = 0.033).

### Prognostic factors for overall survival

Univariate analysis showed that factors affecting OS were maximum tumor size > 5 cm, intraoperative transfusion, cirrhosis, bile duct invasion, lymph node involvement, and vascular invasion. Multivariate analysis showed that cirrhosis was an independent prognostic factor for poor OS (hazard ratio 2.072, 95% confidence interval 1.041–4.123; *P* = 0.038) (Table 4).

**Table 4:** Multivariate analysis of risk factors for poor overall survival

Variables	HR	95% CI	*P*-value
Maximum tumor size > 5 cm	2.115	0.901–4.960	0.085
Intraoperative transfusion	1.704	1.062–2.732	0.027
Cirrhosis	2.072	1.041–4.123	0.038
Bile duct invasion	1.662	0.614–4.511	0.317
Lymph node involvement	1.943	0.829–4.490	0.126
Vascular invasion	2.583	1.380–4.834	0.002

*HR* hazard ratio; *CI* confidence interval

## Discussion

The reported prevalence of cirrhosis in cHCC-CC patients ranges widely from 27.7% to 84.6% worldwide based on operative findings [6]. This figure is 63.2% in our cohort. The sex ratio of cHCC-CC shows a prominent male predominance, which is compatible with the findings of several previous reports [2–5]. It has been reported that this male predominance correlated with high activities of androgen axis, an oncogenic pathway involved in hepatocarcinogenesis [9]. However, further analysis of the precise mechanisms for male susceptibility to cHCC-CC is needed.

cHCC-CC is reportedly similar to HCC in terms of clinicopathologic characteristics including mean age, male/female ratio, hepatitis viral positivity, serum AFP level, and the presence of cirrhosis [1]. Some researchers from Asian institutions therefore speculated that cHCC-CC represents a variant of ordinary HCC that exhibits cholangiocellular metaplasia, rather than a true intermediate disease entity between HCC and CC [3]. As is the case with HCC, we find that hepatitis B virus (HBV) is a main etiologic factor in the development of cHCC-CC in a cirrhotic liver. Accordingly, ALT and ALT values as indicators of activity or severity of the hepatitis state were both higher in cirrhotic patients than those in non-cirrhotic patients. A comparison of the pathologic findings in resected specimens showed the tumor size was generally smaller in cirrhotic group. One possible explanation for this phenomenon is that cirrhotic patients generally have active liver disease and may have image-based liver screening, which enabled detection of small tumors. However, it should be acknowledged that there may be a selection bias for hepatic resection. Many cirrhotic patients were unable to undergo hepatectomy because of poor liver function reserve, and most patients with large tumors may be treated by a nonsurgical modality such as hepatic artery embolization or conservative treatment.

As expected, cirrhotic patients had a significantly higher incidence of liver dysfunction after surgical resection. As cirrhotic patients have relatively small tumours and limited hepatic functional reserve, they usually undergo minor hepatectomy.

The negative impact of cirrhosis on long-term survival has been reported in postoperative HCC patients [10, 11], but its impact on long-term survival of cHCC-CC patients undergoing hepatectomy remains unclear. The present study is the first to present data to indicate that the cirrhosis is an independent predictor for postoperative OS of cHCC-CC patients. The 5-year OS rate was 34.5% in cirrhotic patients *versus* 54.1% in non-cirrhotic counterparts. This difference is likely attributable to more hepatic decompensation caused by ongoing cirrhosis itself in cirrhotic patients. As demonstrated in our study, hepatic insufficiency-related death accounted for 11 (12.1%) deaths in cirrhotic patients and only one (1.9%) death in non-cirrhotic patients. Difference in treatment strategies for recurrent disease may also account for differences in outcomes. Cirrhotic patients usually have impaired hepatic function after the initial hepatic resection, which limits the application of aggressive management for recurrence, which is often the leading cause for an unfavorable outcome.

Several studies have documented an association between cirrhosis and recurrence of HCC, which is likely attributable to multicentric *de novo* carcinogenesis in the remnant liver [10, 12]. However, our study failed to find such an association in cHCC-CC patients. One of the explanations for this discrepancy is that cHCC-CC with CC components exhibits a more aggressive behavior and has high probability of intrahepatic metastasis, which would overshadow the effect of cirrhotic liver related-carcinogenesis.

Theoretically, liver transplantation (LT) offers the potential benefit of resecting the entire tumor-bearing liver and eliminating cirrhosis simultaneously, and therefore it is generally believed to be an ideal approach for the treatment of cHCC-CC in cirrhotic patients. In the three cHCC-CC patients receiving LT reported by Chan et al. [13], one patient died from distant metastasis 16.5 months after operation while the other two patients survived 25 and 35 months after operation, respectively. Wu et al. [14] reported a 5-year OS rate of 39% in a case series of 21 patients with cHCC-CC treated with LT. Panjala et al. [15] reported a 5-year OS rate of 16% in their 12 cHCC-CC patients receiving LT. Employing the Surveillance, Epidemiology, and End Results database (1988–2009), Garancini et al. [16] reported a 5-year OS rate of 41.1% in 16 cHCC-CC patients receiving LT. Currently, it is difficult to assess the effectiveness of LT in the management of cHCC-CC because of insufficient data and limited evidence available.

## Conclusion

This study showed that cHCC-CC patients with cirrhosis had a poorer long-term prognosis after surgical resection as compared with those without cirrhosis, possibly due to the decreased liver function.

## Abbreviations

AFP: Alpha-fetoprotein
ALT: Alanine aminotransferase
AST: Aspartate aminotransferase
CC: Cholangiocarcinoma
cHCC-CC: Hepatocellular-cholangiocarcinoma
CI: Confidence interval
DFS: Disease-free surviva
HBsAg: Hepatitis B surface antigen
HBV: Hepatitis B virus
HCC: Hepatocellular carcinoma
HR: Hazard ratio
LT: Liver transplantation
OS: Overall survival
PRFA: Percutaneous radiofrequency ablation
TACE: Transarterial chemoembolization
